# The HKA axis varies significantly with knee motion: A robot‐assisted intraoperative evaluation during total knee arthroplasty supports the use of dynamic, not static, alignment classifications

**DOI:** 10.1002/jeo2.70370

**Published:** 2025-07-18

**Authors:** Fjorela Qordja, Pieralberto Valpiana, Luca Andriollo, Stefano Marco Paolo Rossi, Andrea Giordano Salvi, Guido Bocchino, Karlos Zepeda, Francesco Benazzo, Pier Francesco Indelli

**Affiliations:** ^1^ Department of Clinical and Molecular Sciences, Clinic of Orthopaedics Università Politecnica delle Marche Ancona Italy; ^2^ Südtiroler Sanitätsbetrieb Brixen Italy; ^3^ Institute of Biomechanics, Paracelsus Medical University (PMU) Salzburg Austria; ^4^ Department of Orthopedics Catholic University of the Sacred Heart Rome Italy; ^5^ Robotic Arthroplasty Unit, Department of Orthopaedics and Traumatology Fondazione Poliambulanza Brescia Italy; ^6^ Department of Life Science, Health, and Health Professions Università degli Studi Link Roma Italy; ^7^ Hospital for Special Surgery, Adult Reconstruction and Joint Replacement Service New York New York USA; ^8^ IUSS Istituto Universitario di Studi Superiori Pavia Italy; ^9^ The Breyer Center for Overseas Studies Stanford University in Florence Florence Italy

**Keywords:** alignment, dynamic HKA, hip–knee–ankle angle, HKA, TKA

## Abstract

**Purpose:**

New alignment classifications based on phenotype reproduction have recently been introduced in total knee arthroplasty (TKA) as alternatives to traditional mechanical alignment. These classifications were designed according to the static hip–knee–ankle angle (sHKA) measurement from long leg radiographs (LLRs). This study aimed to understand whether and how the HKA varied throughout the knee's range of motion (ROM) during robot‐assisted TKA.

**Methods:**

This prospective, bi‐centric cohort study involved 107 consecutive patients undergoing primary robot‐assisted TKA. The surgical technique adhered to restricted kinematic alignment (HKA ± 3°) with asymmetric gap balancing principles. The HKA's dynamic variation (dHKA) was assessed intraoperatively at full extension, as well as at 30°, 45°, 60°, 90° and 120°, both before bone cuts and after the positioning of the trial components. The overall cohort was initially analyzed, followed by a subgroup analysis based on varus, neutral and valgus phenotypes. A descriptive analysis was conducted to evaluate dHKA trends. Collected data were then analyzed using one‐way repeated measures analysis of variance with Bonferroni correction and Bland–Altman plots to assess significant variations in dHKA across the ROM during flexion and to quantify outliers from the established safe boundaries of ±3°.

**Results:**

Out of 107 knees, the pre‐cut dHKA demonstrated a biphasic trend, decreasing in varus until 60° and then transitioning toward valgus, with significant differences primarily noted at 90° and 120°. Post‐cut, the dHKA exhibited an overall varus trend, increasing from full extension to 60° before experiencing a partial recovery. Significant differences were detected primarily at the initial flexion angles. Outlier rates increased with flexion: pre‐cut from 6.5% to 43.0%, and post‐cut from 1.9% to 30.8%, highlighting progressive inter‐individual variability throughout. Although the analysis was stratified by knee phenotype, the post‐cut dHKA trend did not differ among the various phenotypes or in comparison to the overall cohort trend.

**Conclusions:**

The main finding of the current study was that intraoperative dHKA differs significantly from sHKA during robot‐assisted TKA. Moreover, the sHKA was limited in predicting the actual kinematic HKA. Planning the final TKA alignment on static, standing LLRs may have limited value compared to intraoperative planning conducted with enabling technologies.

**Level of Evidence:**

Level 3.

**Abbreviations**
BMI: body mass index
CPAK: coronal plane alignment of the knee
CT: computed tomography
dHKA: dynamic hip–knee–ankle
HKA: hip–knee–ankle
IRB: institutional review board
KA: kinematic alignment
LLR: long‐standing radiography
MA: mechanical alignment
MC: medial congruent
OA: osteoarthritis
ROM: range of motion
SD: standard deviation
sHKA: static hip–knee–ankle
TKA: total knee arthroplasty

## INTRODUCTION

The high dissatisfaction rate among total knee arthroplasty (TKA) patients has recently prompted the introduction of alternatives to the traditional mechanical alignment (MA) approach [28, 40]. In fact, although MA TKAs showed excellent long‐term survivorship results, this technique clearly modifies the native knee anatomy [1]. In search of a more anatomically respectful solution, a few pioneers introduced the kinematic alignment (KA) technique to restore the native tibial‐femoral articular surface along its native rotational axes [23]. However, several modern personalized knee arthroplasty techniques have evolved over the last decade from the original KA [14, 26, 47]. These techniques aimed to preserve the KA philosophy while adhering to alignment boundaries to avoid producing extreme anatomies that could jeopardize implant survivorship and overall functional outcomes [36, 39, 42, 44].

The definition of knee alignment in TKA relies on measuring the hip–knee–ankle (HKA) axis [15], which corresponds to the angle subtended by the mechanical axes of the femur and tibia. It is typically measured using digital long leg radiographs (LLRs) or computed tomography (CT) scans [29], representing a static modality (sHKA). A CT‐based study reported that the HKA ranged from 24° varus to 25° valgus in patients scheduled for a TKA [1]. The current authors have routinely applied KA principles to their robot‐assisted TKA procedures, establishing alignment boundaries based on intraoperative sHKA determination [39].

Several authors, including KA pioneers, analyzed gait in healthy subjects and TKA patients, highlighting that the sHKA, as measured on LLRs, differs significantly from the dynamic HKA (dHKA) during the gait cycle and that dHKA may better predict knee loading during level walking compared to sHKA [6, 34]. Interestingly, the literature lacks studies correlating coronal alignment with TKA survivorship, even among HKA outliers in MA TKA [5, 20, 22]. Little is also known about how the variability of knee coronal alignment throughout the gait cycle affects implant survivorship and, most importantly, whether surgeons should plan the alignment of their TKA based on static or dynamic HKA.

Given the rising interest in personalized alignment and the limitations of static assessments, a deeper understanding of how HKA behaves dynamically throughout the full range of motion (ROM) is essential. To date, few studies have assessed intraoperative dHKA across multiple flexion angles using robotic‐assisted technology. Furthermore, it remains unclear whether current ‘safe zones’ based on static alignment are suitable when accounting for dynamic changes during flexion. This study addresses this gap by analyzing intraoperative coronal alignment during knee flexion, both before and after bone resections, to determine whether static alignment measurements accurately represent the dynamic behaviour of the knee.

This study aimed to understand whether and how the HKA varies throughout the knee ROM, utilizing a novel application of robot‐assisted technology. The authors hypothesized that no significant differences would exist in HKA measured at various knee flexion angles compared to the sHKA measured intraoperatively at full extension.

## METHODS

### Study design and setting

This was a prospective, bi‐centric cohort study conducted between October 2024 and April 2025 at Ospedale di Bressanone and Fondazione Poliambulanza Istituto Ospedaliero. The study enroled consecutive patients affected by primary knee osteoarthritis (OA) who were undergoing primary TKA. All patients received a cruciate‐retaining Persona implant (Zimmer Biomet) with a medial congruent (MC) insert. All procedures were performed using the ROSA® robotic‐assisted surgical system (Zimmer Biomet).

### Inclusion and exclusion criteria

The inclusion criteria were a diagnosis of primary terminal knee OA, an age range of 18–80 years, ROSA® robotic‐assisted TKA (Zimmer Biomet), MC insert and patients' consent to participate. Analysis was first conducted on the entire cohort, followed by stratification into three subgroups based on coronal alignment at dHKA at full extension: varus (dHKA < 177°), neutral (177° ≤ dHKA ≤ 183°) and valgus (dHKA > 183°) [4, 33].

Patients were excluded if they had post‐traumatic knee OA due to previous diaphyseal femoral or tibial fractures, abnormal ligament laxity, a previous ipsilateral total hip replacement, a different degree of constraint, or in cases of revision TKA.

### Surgical procedure

In all cases, a standard medial parapatellar approach was utilized. The pins for the robotic trackers were inserted intraarticularly on the femoral side and extra‐incisional on the tibial side. Following the landmarking steps, osteophyte removal and the sacrifice of the anterior and posterior cruciate ligaments, the evaluation of sHKA (with the knee in maximum extension as guided by gravity) and dHKA was carried out throughout the knee flexion arc, paying maximum attention to avoid any frontal plane stress.

These measurements have always been performed by two examiners (PV and SMPR) to limit variability in data acquisition: the examiners were able to limit any varus/valgus stress on the knee, but the physiological external rotation of the femur and abduction at the hip joint were not limited, as their role in HKA variability has been demonstrated [45]. Unfortunately, during the dHKA recordings, the examiners could not reposition the extensor mechanism due to the intraarticular presence of the robotic trackers' pins. Notably, dHKA values were collected at full extension and knee flexions of 30°, 45°, 60°, 90° and 120°. The bone cuts were planned according to a modern personalized arthroplasty technique described in prior studies [35, 39].

The intraoperative goal was always to resurface the tibiofemoral joint, following a modification of an algorithm proposed by Vendittoli et al. and Van‐Essen et al. [39, 40] that balanced the soft tissue envelope to create a slightly asymmetric intercompartmental gap (*Δ* > 2 mm in extension or *Δ* > 4 mm in flexion) while remaining within a safe alignment zone (MPTA and LDFA 0 ± 5°, HKA 0 ± 3°).

If a significant intercompartmental imbalance was detected (*Δ* > 2 mm in extension or *Δ* > 4 mm in flexion), the surgical plan was adjusted to respect those soft tissue safe boundaries. After the robot‐assisted bone cuts, cruciate‐retaining femoral, tibial and MC Persona® (Zimmer Biomet) trial components were inserted into place. A classical stability check was then conducted throughout the entire ROM. At this point, dHKA was reevaluated throughout the knee flexion arc, and final measurements were collected at full extension and knee flexions of 30°, 45°, 60°, 90° and 120°. Afterwards, pins were removed, and the knee joint was closed in a standard manner.

### Demographic and radiographic data

The recorded demographic data included sex, age at surgery, weight, body mass index (BMI) and knee laterality. According to the radiographic assessment, all patients underwent a preoperative standing LLRs to calculate the preoperative sHKA (pre‐sHKA). At 6 weeks post‐operatively, patients repeated the LLRs to reassess the post‐operative sHKA (post‐sHKA). Two observers (FQ and GB) independently evaluated all radiographic measurements.

### Statistical analysis

Data were collected and organized using Microsoft Excel (Microsoft Corp.). Statistical analysis was conducted using SPSS 26 (IBM Corp.).

The normality of the continuous variable was confirmed using the Shapiro–Wilk test. The continuous variables were reported as mean ± standard deviation (SD), while categorical variables were reported as absolute frequencies and percentages. A one‐way repeated measures analysis of variance (ANOVA) was employed to evaluate within‐subject variability of the dHKA across different flexion angles. When significant, a Bonferroni post hoc test was utilized to identify specific angle comparisons contributing to the overall effect.

Additionally, the relationship between the sHKA measured on preoperative LLRs and the intraoperative dHKA recorded in full extension before the bone cuts was analyzed. This comparison was repeated after the trial component positioning. To assess significance, a paired t‐test was conducted.

A Bland–Altman plot was constructed to evaluate the level of agreement of the dHKA measurements across the ROM relative to full extension. A threshold of ±3° from the plot's bias was used to define clinically relevant outliers. These analyses were conducted separately for both the pre‐cut and post‐cut settings, with significance set at an *α* value of 0.05.

An a priori power analysis for one‐way repeated measures ANOVA was conducted. Using a power of 80%, an *α* of <0.05, and estimating a low effect size of 0.1, a minimum of 99 patients is required for pairwise comparison.

The study obtained approval from the institutional review board (IRB: SABES 71/2023) and was conducted in accordance with the Helsinki Declaration.

## RESULTS

One hundred seven subjects met the inclusion criteria and were recruited for this study. The majority of cases involved varus knees, accounting for 62.6% (67/107), followed by neutral knees at 29.9% (32/107), while valgus knees represented the least frequent group at 7.5% (8/107).

### Demographic and radiological results

The mean age of the 107 patients included in the study was 72 ± 9.3 years, with a female predominance (55% vs. 45%). The right knee was operated on in 59 cases, compared to 48 left knees. The mean BMI was 29.9 ± 5.3, as shown in Table 1.

**Table 1:** Patients' demographic and radiological characteristics.

Variables	Values
Age (mean ± SD)	72 ± 9.31
Sex	
Male (%)	48 (45%)
Female (%)	59 (55%)
Weight (kg)	86.7 ± 18.27
BMI (kg/m^2^)	29.9 ± 5.33
Laterality	
Left (%)	48 (45%)
Right (%)	59 (55%)
Pre‐sHKA (mean ± SD)	176 ± 5.95
Post‐sHKA (mean ± SD)	178.6 ± 3.06

Abbreviations: BMI, body mass index; SD, standard deviation; sHKA, static hip–knee–ankle.

The mean sHKA on preoperative LLRs was 176.0 ± 5.95°, increasing to 178.6 ± 3.06° on post‐operative films. The dHKA used for comparison was recorded intraoperatively in full extension, both before bone cuts and after trial component positioning. The mean difference between pre‐cut dHKA at full extension and preoperative sHKA measured on LLRs was −0.08 ± 3.82°, with no statistically significant difference found via the paired *t* test (*p* = 0.926). In contrast, the post‐cut dHKA at full extension significantly differed from post‐operative sHKA (*p* = 0.027), with a mean difference of −0.63 ± 2.68°.

### Comprehensive description of the overall cohort

Table 2 summarizes the main results observed in the overall cohort regarding dHKA variation and the percentage of outliers.

**Table 2:** Overall cohort dHKA trend and outlier's percentage according to Bland–Altman plots.

Variable	Pre‐cut		Post‐cut	
	dHKA variation, mean ± SD (°)	Bland–Altman outliers, *n* (%)	dHKA variation, mean ± SD (°)	Bland–Altman outliers, *n* (%)
dHKA_full extension_	176.0 ± 4.10	‐	176.1 ± 3.73	‐
dHKA_30°_	175.6 ± 4.09	7 (6.5)	175.6 ± 3.72	2 (1.9)
dHKA_45°_	175.5 ± 4.22	18 (16.8)	175.2 ± 3.76	10 (9.4)
dHKA_60°_	175.5 ± 4.35	21 (19.6)	175.1 ± 3.80	20 (18.7)
dHKA_90°_	176.1 ± 4.45	35 (32.7)	175.4 ± 3.84	34 (31.8)
dHKA_120°_	177.0 ± 3.37	46 (43.0)	175.8 ± 4.24	33 (30.8)

Abbreviations: dHKA, dynamic hip–knee–ankle; n, number; SD, standard deviation.

#### Pre‐cut intraoperative results

**Figure 1:**
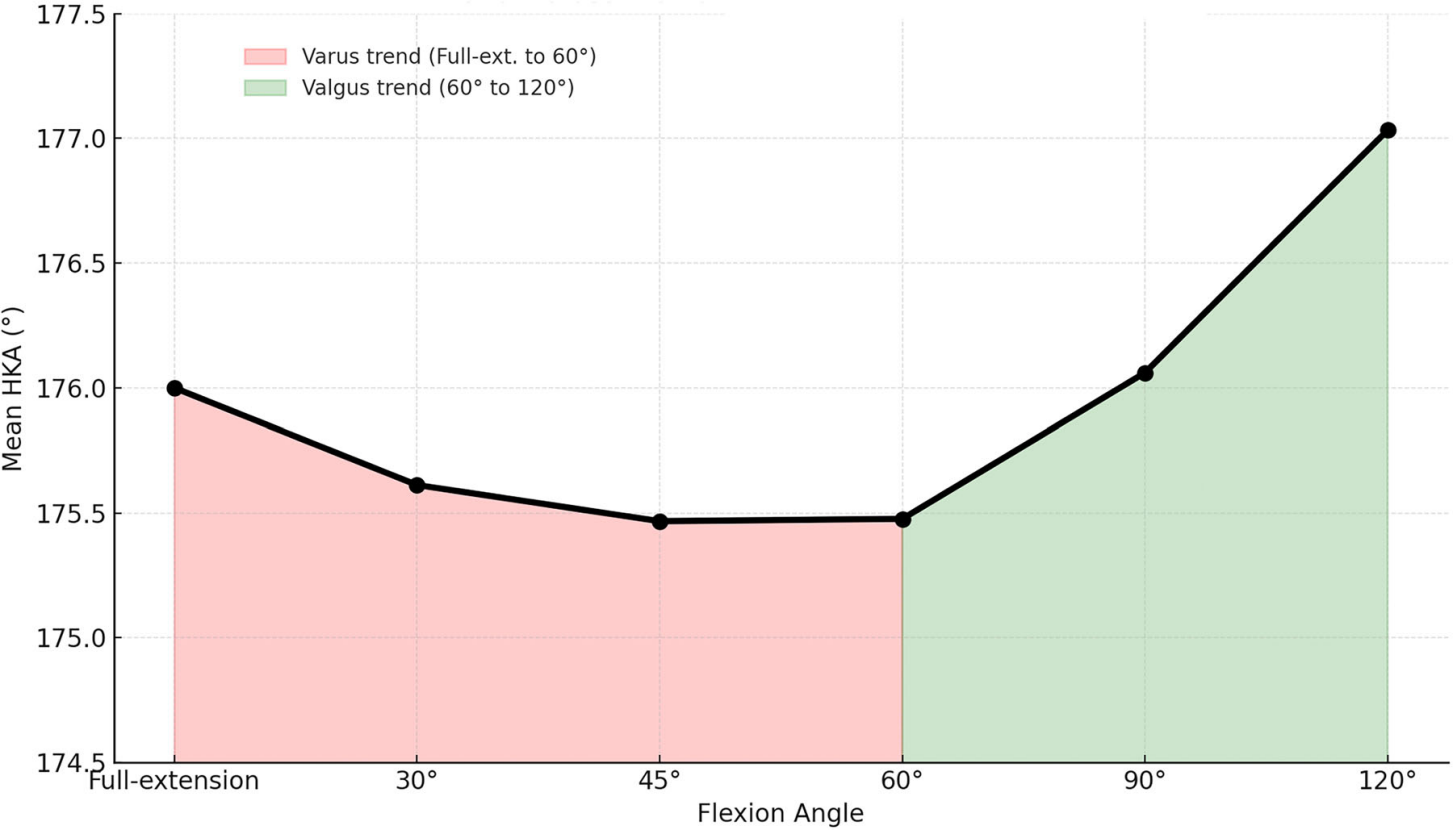
Pre‐cuts dHKA trend across flexion: Varus to valgus shift. dHKA, dynamic hip–knee–ankle.

**Figure 2:**
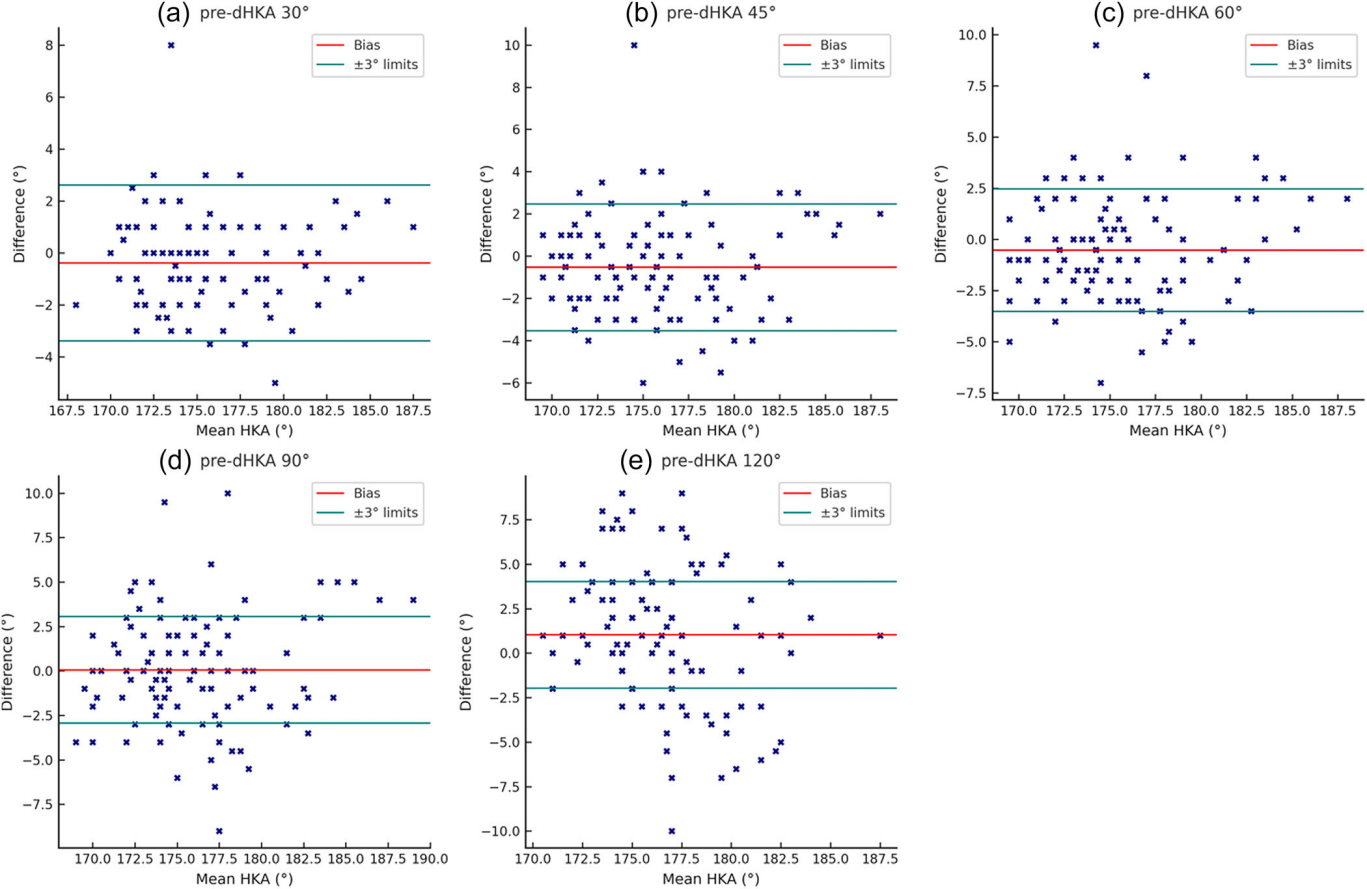
Pre‐cuts dHKA outliers from the safe limits ±3° during arch of motion: (a) 6.5% at 30°; (b) 16.8% at 45°; (c) 19.6% at 60°; (d) 32.7% at 90°; (e) 43.0% at 120°. dHKA, dynamic hip–knee–ankle.

First, the overall cohort showed a biphasic pattern based on coronal alignment. The mean dHKA displayed a decreasing trend up to 60°, indicating a progressive varus shift. Beyond 60°, the trend reversed, exhibiting valgus behaviour (Figure 1). Multiple comparisons using one‐way ANOVA confirmed a statistically significant effect of joint angle on dHKA measurements (*F*(2.45, 259.72) = 11.28, *p* < 0.01). The post hoc Bonferroni correction revealed a non‐linear, flexion‐dependent change in the dHKA, with the most significant deviation occurring between mid‐ and deep‐flexion. In particular, dHKA values at 120° were significantly different from all other positions (*p* < 0.01). Additionally, a significant difference was also noted between 60° and 90° (*p* < 0.01). In contrast, no meaningful deviation was observed between full extension and any flexion position. Concerning outliers, the Bland–Altman pre‐cut plots showed that the proportion of outliers increased progressively with higher degrees of flexion, reaching the highest percentage at 90° (Figure 2).

#### Post‐cut intraoperative results

**Figure 3:**
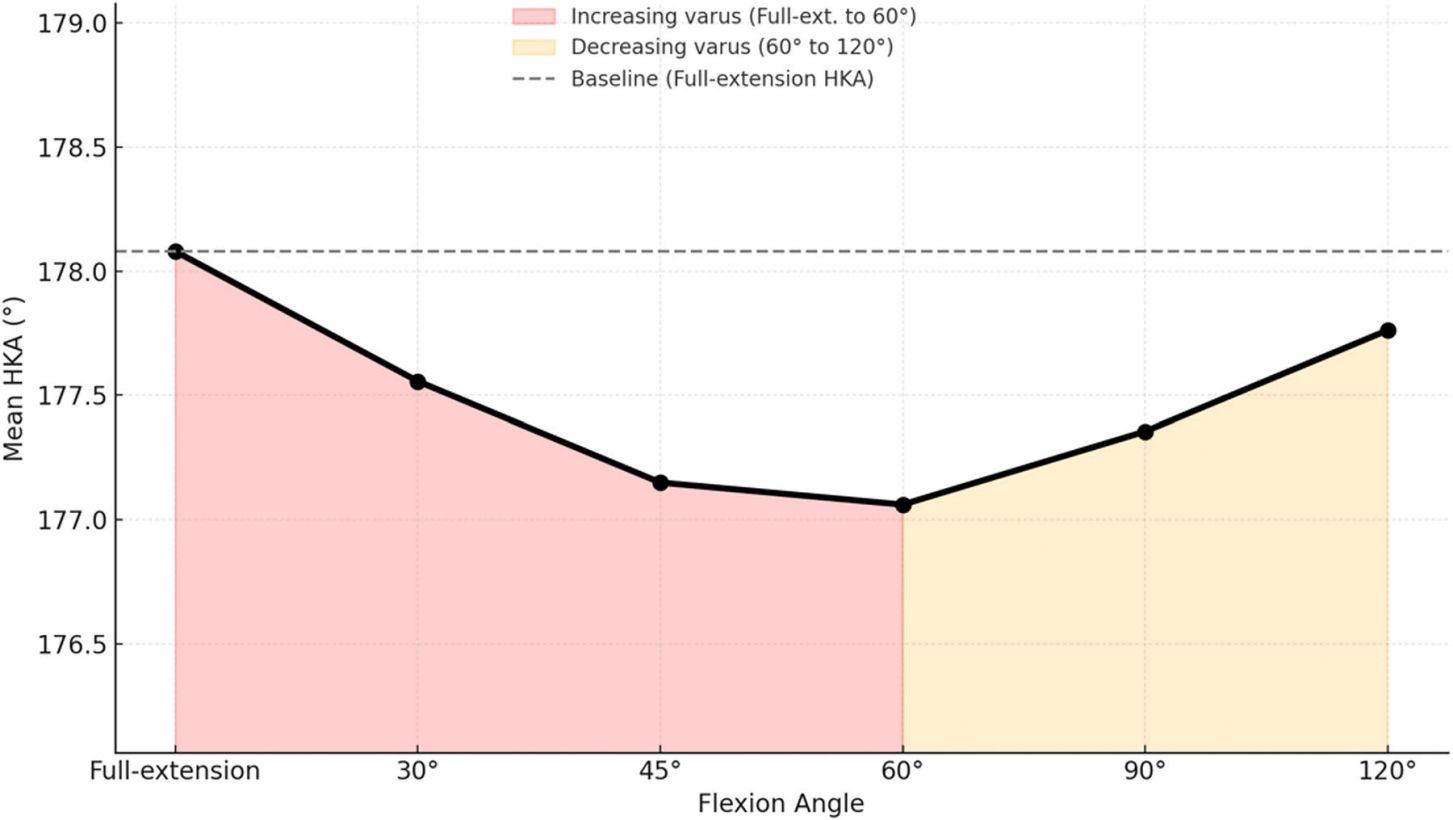
Post‐cuts dHKA trend across flexion: varus trend with partial recovery. dHKA, dynamic hip–knee–ankle.

**Figure 4:**
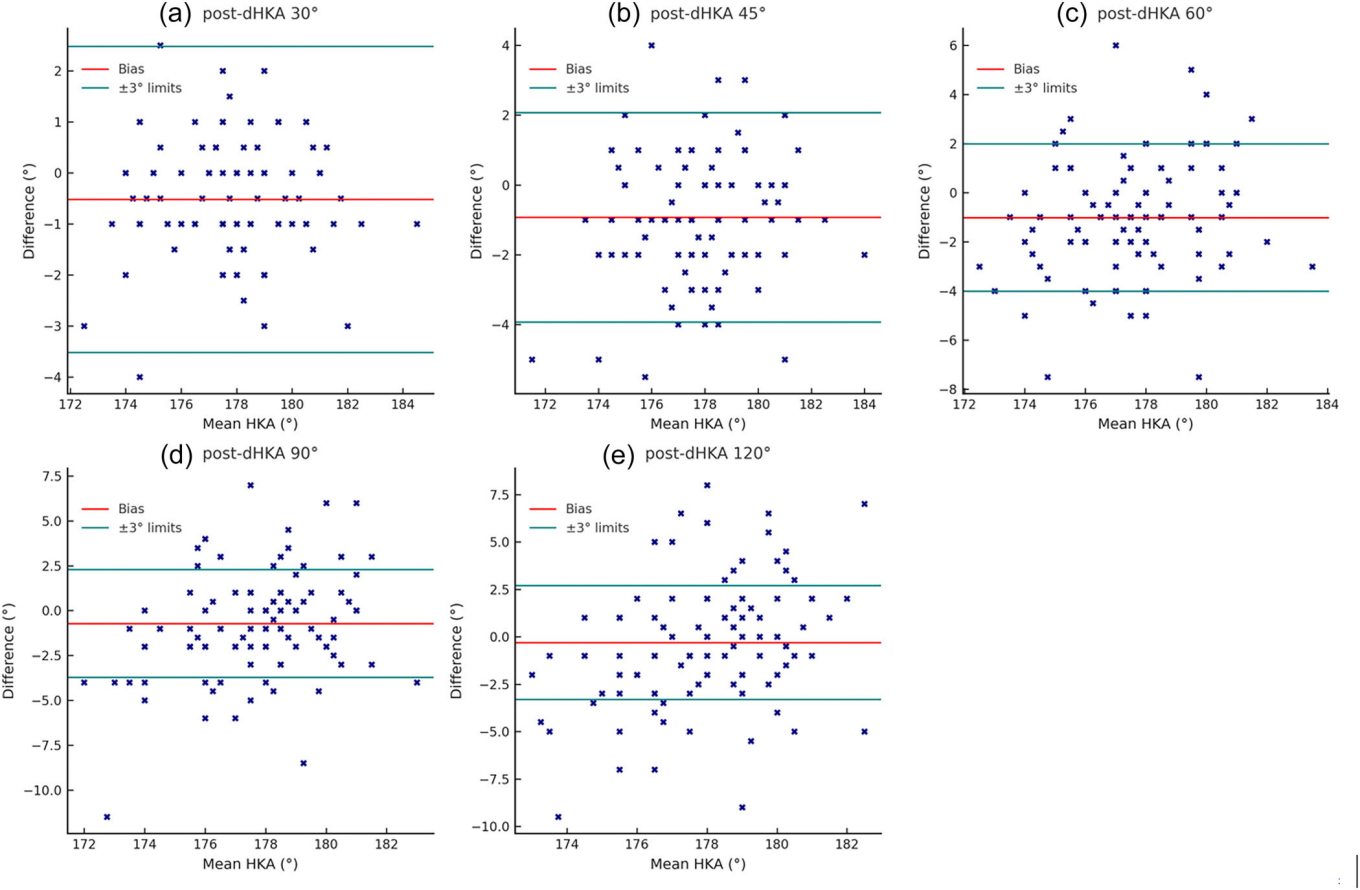
Post‐cuts dHKA outliers from the safe limits of ±3° throughout the arc of motion: (a) 1.9% at 30°; (b) 9.4% at 45°; (c) 18.7% at 60°; (d) 31.8% at 90°; (e) 30.8% at 120°. dHKA, dynamic hip–knee–ankle.

Following trial component positioning, a progressive varus trend in the dHKA's mean values was observed from full extension to mid flexion, with a partial reduction of varus noted in deeper flexion (Figure 3). The one‐way repeated measures ANOVA demonstrated a statistically significant effect of flexion angle on dHKA (*F*(5, 530) = 7.58, *p* < 0.01). Post‐cut Bonferroni correction revealed significant differences between full‐extension and early flexion positions, specifically for 0° versus 30° (*p* < 0.01), 0° versus 45° (*p* < 0.01), and 0° versus 60° (*p* < 0.01). Additionally, dHKA differed significantly between 30° and 45° (*p* < 0.01) as well as between 60° and 120° (*p* < 0.01). Finally, the persistence of inter‐individual variability was confirmed by Bland–Altman analysis of outliers (Figure 4).

### Stratified‐cohort results

Table 3 shows the dHKA values throughout the complete ROM for the varus, neutral and valgus knees of the cohort. Table 4 summarizes the number of outliers exceeding the safety threshold at various degrees of flexion‐extension for all three analytical subgroups, both before and after prosthetic implantation of the native knee.

**Table 3:** dHKA trend among varus, neutral and valgus knee cohorts.

Variable	Varus		Neutral		Valgus	
	Pre‐cut (mean ± SD)	Post‐cut (mean ± SD)	Pre‐cut (mean ± SD)	Post‐cut (mean ± SD)	Pre‐cut (mean ± SD)	Post‐cut (mean ± SD)
dHKA_full extension_	173.38 ± 2.05	177.11 ± 1.89	178.16 ± 5.19	179.28 ± 1.62	184.25 ± 1.41	181.38 ± 1.53
dHKA_30°_	173.22 ± 2.21	176.59 ± 1.96	178.45 ± 2.39	178.80 ± 1.75	184.25 ± 2.38	180.69 ± 1.44
dHKA_45°_	173.25 ± 2.51	176.25 ± 2.11	177.94 ± 2.92	178.28 ± 2.26	184.19 ± 3.35	180.19 ± 1.73
dHKA_60°_	173.28 ± 2.68	176.22 ± 2.30	177.92 ± 3.34	178.22 ± 2.55	184.13 ± 3.26	179.44 ± 1.64
dHKA_90°_	174.04 ± 2.92	176.57 ± 2.63	178.19 ± 3.73	178.45 ± 3.26	184.00 ± 4.01	179.50 ± 1.41
dHKA_120°_	176.01 ± 2.78	177.04 ± 2.73	178.31 ± 3.40	178.72 ± 3.48	180.50 ± 4.04	180.00 ± 0.96

Abbreviations: dHKA, dynamic hip–knee–ankle; SD, standard deviation.

**Table 4:** Outliers for varus, neutral and valgus knees according to Bland–Altman plots.

Variable	Varus knee		Neutral knee		Valgus knee	
	Pre‐cut outliers, *n* (%)	Post‐cut outliers, *n* (%)	Pre‐cut outliers, *n* (%)	Post‐cut outliers, *n* (%)	Pre‐cut outliers, *n* (%)	Post‐cut outliers, *n* (%)
dHKA_30°_	4 (6.09)	2 (3.0)	1 (3.1)	0 (0)	0 (0)	0 (0)
dHKA_45°_	8 (11.9)	5 (7.5)	7 (21.9)	4 (12.5)	1 (12.5)	0 (0)
dHKA_60°_	10 (14.9)	12 (17.9)	11 (34.4)	8 (25.0)	2 (25.0)	0 (0)
dHKA_90°_	16 (23.9)	22 (32.8)	14 (43.8)	11 (34.4)	5 (62.5)	0 (0)
dHKA_120°_	21 (31.3)	17 (25.4)	10 (31.2)	15 (46.9)	3 (37.5)	1 (12.5)

Abbreviations: dHKA, dynamic hip–knee–ankle; n, number.

#### Varus knee cohort

On average, the native varus knees demonstrated an increasing varus trend up to 60°, followed by a shift toward valgus at higher flexion angles (Table 3). After the trial positioning, the dHKA displayed a varus trend with partial correction beyond 60°. ANOVA revealed a statistically significant overall variation across flexion‐extension angles in the pre‐cut setting (*F*(6, 422) = 10.67, *p* < 0.001), but not in the post‐cut condition (*F*(5, 396) = 1.83, *p* = 0.107). No individual angle remained significant after Bonferroni correction. In both conditions, the number of outliers progressively increased with knee flexion, peaking at 90° and 120° (Table 4).

#### Neutral knee cohort

The neutral knee subgroup displayed a varus‐oriented pattern with partial correction beyond 60°, both in the native condition and after trial component placement (Table 3). Repeated‐measures ANOVA revealed no significant variation in either the pre‐cut (*F*(6, 196) = 1.02, *p* = 0.414) or post‐cut phase (F(6, 196) = 0.66, *p* = 0.684). Between 90° and 120°, nearly half of the knees exceeded the ±3° safety threshold (Table 4).

#### Valgus knee cohort

A progressive shift towards varus characterized the valgus OA knee cohort; between 90° and 120°, the mean dHKA decreased by 3.75°. Prosthetic bone resections reduced the mean absolute dHKA values at all flexion angles, resulting in partial correction of the varus deviation beyond 60° (Table 3). ANOVA did not reveal any significant variation in dHKA within this group. The pre‐cut valgus subgroup showed the highest percentage of outliers across the entire cohort; following trial component placement, outliers were nearly absent (Table 4).

## DISCUSSION

The primary finding of the current study was that, in robot‐assisted TKA, the intraoperative HKA varies significantly during passive ROM evaluation, both before and after the bone cuts. The authors demonstrated a more substantial difference between the sHKA and the dHKA in two distinct scenarios: in the native knee, from mid‐flexion to full flexion, and with the trial components in place, between maximum extension and mid‐flexion. Consequently, the sHKA, which represents the target of many TKA alignment classifications [16, 24, 42, 44], may have limited value in estimating the dynamic loading that occurs during gait, as it is traditionally measured in full extension.

The quest for more personalized knee alignment surgical techniques has created an epidemic of TKA alignment classifications. Lin et al. [24] reviewed LLRs of 214 native knees and described 27 possible phenotypes, but only considered five clinically relevant. Hirschmann et al. [17] studied CT scans of 380 native knees, revealing 125 possible phenotypes, but only deemed 43 clinically relevant. MacDessi et al. [27] reviewed digital LLRs from 500 healthy knees and 500 osteoarthritic knees, describing nine categories of coronal knee alignment (coronal plane alignment of the knee [CPAK]). Unfortunately, using static, weight‐bearing films has well‐known advantages in determining the degree of cartilage wear [8] but little value in predicting the actual dHKA during the gait cycle in healthy and osteoarthritic patients [46].

The current authors have already questioned the epidemic of static classifications in TKA alignment [19], advocating for a more dynamic approach. Few authors have supported the use of gait analysis platforms to perform knee coronal alignment measurements throughout the gait cycle [6, 7, 13, 18], suggesting that the dHKA could potentially reflect knee loading more accurately and, therefore, be more predictive of long‐term TKA survivorship outcomes.

Nevertheless, many studies have shown limitations inherent in the gait analysis setup, such as issues with soft tissue artefacts and the inability to analyze other activities, such as running or jumping, which require greater flexion angles than those during level walking tasks [6, 13, 34].

A recent study by Andriollo et al. [3] found that an HKA at 90° of flexion after TKA of ≥5° varus demonstrated superior Knee Society Score function and knee scores compared to those with a final HKA at 90° of flexion between 0° and 4° varus. Furthermore, patients with intraoperative changes in HKA at 90° of flexion >2.5° towards neutralization (varus reduction or a shift towards valgus) achieved better Forgotten Joint Score and Anterior Knee Pain scores.

The recent quest for more personalized knee alignment has created a need for greater accuracy during intra‐operative TKA steps, thereby supporting the adoption of computer navigation and robotic‐assisted surgery [36]. Among multiple options, imageless systems have recently gained popularity due to their accuracy [25], user‐friendly characteristics [39], precision in landmarking compared to standard radiographs [9] and reliability that is non‐inferior to CT‐scan‐based systems [14]. Moreover, recent concerns about additional radiation risks from adjunctive CT scans may support the use of imageless systems instead of image‐based ones [37].

The robotic system utilized in the current study exhibits several characteristics: it plans bone cuts according to the surgeon's alignment preferences, allows for dynamic balancing of the knee in both extension and flexion, and ultimately, can detect the HKA intraoperatively throughout the entire ROM. The authors employed this last characteristic to conduct intraoperative knee dynamic analysis, which proved to be more informative than traditional radiological measurements.

Robot‐assisted TKA has been shown to enable the precise reproduction of surgical plans, including accurate determination of coronal limb alignment [31]. Furthermore, unlike biomechanical analysis conducted in a laboratory setting, intra‐operative dynamic analysis benefits from not being affected by soft tissue artefacts, as the trackers are directly attached to the bone. Finally, since many authors proposed modern personalized techniques to set boundaries (i.e., within 3° of a neutral alignment) in the post‐operative alignment of their implants [42] to limit outliers due to the risk of compromising the implant's survivorship [36, 38, 41, 42, 44], the current authors believe that understanding the dynamic variation of HKA could help avoid outliers in TKA alignment.

The correlation between the variability of the dHKA and the clinical findings has yet to be established, representing a significant limitation of the current study. The current study showed that at 30° of knee flexion, the pre‐cuts dHKA fell outside ±3° from the sHKA in 6.5% of the implants; this percentage increased to 32.5% at 90° of flexion and rose to 43.0% at 120°. Interestingly, even after the knee was balanced and the trial components had been positioned, 30% of outliers (those outside ±3° from the sHKA) persisted during deep knee flexion. The authors believe that the high number of outliers reported in this study may threaten the true definition of ‘safe limits’ in TKA alignment [43] What is intended as ‘safe’ at heel strike (knee in full extension) during the gait cycle might be considered not ‘safe’ during other phases of the gait cycle. It has been already established that the most critical gait phases in terms of patient‐reported instability are the early stance phase and the late stance‐to‐early swing phase [10, 32]: during these gait phases, the predicted knee flexion ranges from 20° to 60°, which does not account for other activities of daily living, such as bending and squatting, where knee flexion may exceed 130° [18].

In line with the current results, an industry‐funded study by Higinbotham et al. [13] showed a 4° ± 1.04° HKA intraoperative variability during passive knee flexion. In the same study, the surgical parameters most influential in determining the HKA variability included residual medial laxity, varus‐valgus positioning of the femoral component, tibial slope, varus‐valgus positioning of the tibial component and axial rotation of the femoral component. Similarly, our study demonstrated that the intraoperative degree of knee flexion significantly affects the variability of the dHKA compared to the sHKA.

This study has several limitations. The major one is that the coronal alignment achieved intraoperatively, as measured in this study, differs significantly from the alignment measurable in weight‐bearing situations due to the different dynamic frontal plane kinematics of the limb during activities of daily living [7]. This difference is mainly attributed to the soft tissue envelope of the knee [12], which behaves differently in weight‐bearing and non‐weight‐bearing situations [46]. In fact, Zhang et al. [46] compared sHKA and dHKA in healthy individuals and showed an average difference of 9.1 ± 2.8° during the swing phase of the gait cycle. Further complicating this issue, Glenday et al. [11] used finite element modelling to demonstrate that even minor varus deviations in alignment result in increased micromotion and bone stress in cementless tibial baseplates during gait, highlighting the biomechanical significance of dynamic misalignment.

On the other hand, during the early stance phase, described as the critical phase linked to joint stability/instability [10, 32], knee flexion averages 10° at heel strike and increases to 15–20° during the load acceptance phase [30]. Data acquired intraoperatively during this early ROM may correlate with a weight‐bearing scenario, as described by a few authors [13]. Interestingly, gait analysis studies could not establish a correlation between HKA and the Knee Adduction Moment, which represents a dynamic measure linked to the early loosening of TKA [2].

The current authors also acknowledge the limitation that their intraoperative recordings have been influenced by several factors: the extensor mechanism was not closed during the measurements because of the intraarticular presence of the trackers' pins, and the physiological external rotation of the femur and abduction of the hip joint during flexion may have been affected by the force applied to the knee to prevent any varus/valgus, frontal plane stress.

Another limitation is the use of a robotic system during sHKA data acquisition, which may not accurately replicate the static coronal limb alignment as measured on standing LLRs. A notable constraint of this study is the small sample size of the valgus knee group, which reduces the statistical power and generalizability of findings related to this phenotype, particularly regarding the evaluation of outliers. However, this limited representation also reflects the epidemiological distribution of osteoarthritic deformities in the population undergoing TKA [21]. Finally, the authors evaluated a single, medially congruent implant using a strict TKA alignment philosophy, which may compromise its applicability to other designs and alignment strategies.

## CONCLUSION

The primary finding of this study was that intraoperative dHKA differs significantly from sHKA during robot‐assisted TKA. Future studies should investigate the clinical implications of this variability during weight‐bearing activities. Furthermore, the authors warn orthopaedic surgeons to exercise caution when considering sHKA, as measured in LLRs, as their primary target for alignment during TKA procedures. In an era where many surgeons aim to establish boundaries in implant alignment to reduce the risk of outliers, this study suggests a more dynamic and personalized approach. Prioritizing the soft tissue envelope by determining ligamentous intraoperative boundaries, instead of solely focusing on alignment reproduction through osseous boundaries, may represent the best approach when addressing TKA.

## AUTHOR CONTRIBUTIONS

All authors contributed to the conception and design of the study. Pier Francesco Indelli, Stefano Paolo Rossi, Pieralberto Valpiana, Fjorela Qordja, Guido Bocchino, Luca Andriollo and Karlos Zepeda participated in data collection. Fjorela Qordja and Andrea Giordano Salvi participated in the statistical analysis. Pieralberto Valpiana, Guido Bocchino, Andrea Salvi, Luca Andriollo and Fjorela Qordja participated in the interpretation of the data. Andrea Salvi, Stefano Paolo Rossi and Fjorela Qordja drafted the work. Pier Francesco Indelli and Francesco Benazzo revised the manuscript and approved its publication. All authors reviewed and approved the final version of the manuscript.

## CONFLICT OF INTEREST STATEMENT

Pier Francesco Indelli, Stefano Marco Paolo Rossi and Francesco Benazzo are consultants for medical education for Zimmer Biomet, Warsaw, USA. The remaining authors declare no conflicts of interest.

## ETHICS STATEMENT

This study was conducted following the principles of the Declaration of Helsinki. This study received approval from the Internal Review Board (IRB: SABES 71/2023 and 17/2024). Written consent was obtained from participants in the study.

## Data Availability

The data sets used and/or analyzed during this study are available from the corresponding author upon reasonable request.
